# Commentary: Subvalvular procedures offer hope for better results in tricuspid valve repair

**DOI:** 10.1016/j.xjtc.2021.10.022

**Published:** 2021-10-19

**Authors:** Joseph Lamelas, Ahmed Alnajar

**Affiliations:** Division of Cardiothoracic Surgery, The DeWitt Daughtry Department of Surgery, University of Miami Miller School of Medicine, Miami, Fla

**Figure:**
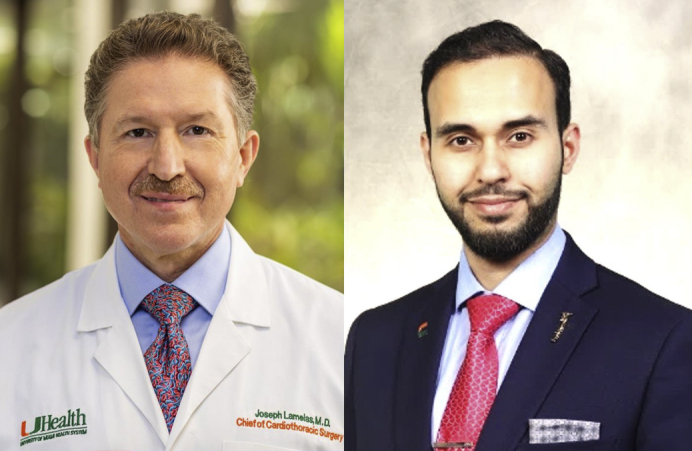
Joseph Lamelas, MD, and Ahmed Alnajar, MD

**Central Message**

TVR may not always be the best option in the setting of functional regurgitation. However, when repair is indicated, concurrent subvalvular procedures could support the use of TVR. As we learn more about combining these procedures, their efficacy will increase and potentially offer more hope for patients in RVF.

See Article page 282.

There is an unmet need to halt the sequelae of right ventricular failure (RVF) and reduce tricuspid valve (TV) deterioration. Left-sided heart failure and pulmonary hypertension result in RVF, triggering in a vicious cycle that leads to increased morbidity and mortality. Due to pulmonary hypertension, volume overload, and/or ischemic wall motion abnormalities in RVF,^1^ the TV papillary muscle is displaced leading to leaflet tethering, which results in functional—or secondary—tricuspid regurgitation. Due to the excess mortality associated with moderate or severe functional tricuspid regurgitation,^2^ TV repair (TVR) may be beneficial. However, annuloplasty alone may fail to meet expectations, leading to recurrent TV regurgitation.^3^^,^^4^ TVR—despite being controversial—became the only viable intervention. Simply leaving severe functional tricuspid regurgitation alone is not an acceptable decision in the long run because conservative options do not result in substantial progress.

Couetil and colleagues^5^ attempted to support evidence of the ability of TVR to change the status quo by performing concurrent papillary muscle septalization. The authors present this novel procedure as a reproducible and simple tool for 11 patients with functional tricuspid regurgitation. The authors corrected tenting of anterior, posterior, and septal TV leaflets. Whereas repeated surgery was required for 2 patients and 2 patients died at 30 days, in such a small study of high-risk patients, the data obtained add to the wealth of promising reports exploring TV subvalvular procedures in adjunct to TVR.^6^, ^7^, ^8^, ^9^, ^10^ Although we may still wonder about the reasons for reoperations and death in the current study, such numbers are often expected when performing similar surgery, and we are remiss to not contextualize such findings. In appropriate clinical scenarios that necessitate thinking outside the box, this technique will be handy, especially in situations where no individual approach has been shown to be superior.

Similar to the case of left ventricular papillary muscle approximation during mitral valve repair,^11^^,^^12^ TVR with papillary muscle septalization has the potential to restore valve competence, improve the degree of ventricular reverse remodeling, increase TVR durability, and reduce New York Heart Association heart failure class, as observed by Couetil and colleagues^5^ in most of their patients within 12 months. Readers should be wary of the inherent limitations of nonrandomized studies with a small sample size and closely observe the mortality and reoperation rates, until a representative randomized control trial provides stronger evidence. However, while we continue debating the best approaches to manage the heterogeneous spectrum of TV diseases, tricuspid subvalvular procedures offer encouragement in the context of functional tricuspid regurgitation to improve right heart function and quality of life.
